# Comparison between Gram stain and culture for the characterization of vaginal microflora: Definition of a distinct grade that resembles grade I microflora and revised categorization of grade I microflora

**DOI:** 10.1186/1471-2180-5-61

**Published:** 2005-10-14

**Authors:** Rita Verhelst, Hans Verstraelen, Geert Claeys, Gerda Verschraegen, Leen Van Simaey, Catharine De Ganck, Ellen De Backer, Marleen Temmerman, Mario Vaneechoutte

**Affiliations:** 1 Department Clinical Chemistry, Microbiology & Immunology, Ghent University Hospital, UGent, Ghent, Belgium; 2 Department of Obstetrics & Gynaecology, Ghent University Hospital, UGent, Ghent, Belgium

## Abstract

**Background:**

The microbiological diagnosis of bacterial vaginosis is usually made using Nugent's criteria, a useful but rather laborious scoring system based on counting bacterial cell types on Gram stained slides of vaginal smears. Ison and Hay have simplified the score system to three categories and added a fourth category for microflora with a predominance of the *Streptococcus *cell type. Because in the Nugent system several cell types are not taken into account for a final score, we carried out a detailed assessment of the composition of the vaginal microflora in relation to standard Gram stain in order the improve the diagnostic value of the Gram stain. To this purpose we compared Gram stain based categorization of vaginal smears with i) species specific PCR for the detection of *Gardnerella vaginalis *and *Atopobium vaginae *and with ii) tDNA-PCR for the identification of most cultivable species.

**Results:**

A total of 515 samples were obtained from 197 pregnant women, of which 403 (78.3%) were categorized as grade I microflora, 46 (8.9%) as grade II, 22 (4.3%) as grade III and 8 (1.6%) as grade IV, according to the criteria of Ison and Hay. Another 36 samples (7.0%) were assigned to the new category 'grade I-like', because of the presence of diphtheroid bacilli cell types. We found that 52.7% of the grade I-like samples contained *Bifidobacterium *spp. while *L. crispatus *was present in only 2.8% of the samples and *G. vaginalis *and *A. vaginae *were virtually absent; in addition, the species diversity of this category was similar to that of grade II specimens.

Based on the presence of different *Lactobacillus *cell types, grade I specimens were further characterized as grade Ia (40.2%), grade Iab (14.9%) and grade Ib (44.9%). We found that this classification was supported by the finding that *L. crispatus *was cultured from respectively 87.0% and 76.7% of grade Ia and Iab specimens while this species was present in only 13.3% of grade Ib specimens, a category in which *L. gasseri *and *L. iners *were predominant.

**Conclusion:**

Further refinement of Gram stain based grading of vaginal smears is possible by distinguishing additional classes within grade I smears (Ia, Iab and Ib) and by adding a separate category, designated grade I-like. A strong correlation was found between grade Ia and the presence of *L. crispatus *and between grade I-like and the presence of bifidobacteria. This refinement of Gram stain based scoring of vaginal smears may be helpful to improve the interpretation of the clinical data in future studies, such as the understanding of response to treatment and recurrence of bacterial vaginosis in some women, and the relationship between bacterial vaginosis and preterm birth.

## Background

Currently the criteria as defined by Nugent *et al*. [1] are considered as the standard procedure to score vaginal smears by Gram stain [2]. This method scores the smears in a standardized manner by quantification of some of the cell types present – designated as *Lactobacillus*, *Gardnerella vaginalis*, *Bacteroides *and *Mobiluncus *'morphotypes'. However, the Nugent scoring system conflates women with potentially very different vaginal microflora in a single category [3]. Since the method requires considerable time and skill, simpler versions have been described which assess the categories in a more qualitative manner [4-6]. Recent developments in our knowledge of the vaginal microflora – including the observation of different *Lactobacillus *species producing different amounts of hydrogen peroxide [7-9] with a potential effect on pregnancy outcome [10,11] urge to refine the Gram stain criteria in an effort to increase the agreement between Gram stain and the true composition of the vaginal microflora. In addition, a strong association of the metronidazole resistant fastidious anaerobic coccobacillus *Atopobium vaginae *with bacterial vaginosis [12-14] might have important implications in the pathophysiology of bacterial vaginosis related preterm labour and birth. The more accurate allocation of subjects according to vaginal microflora status, as assessed by Gram stain, may enhance the validity of studies on the etiology of bacterial vaginosis, and help to better understand response to treatment and recurrence in some women, as well as its relation to preterm birth.

Here we report our findings obtained by studying a total of 515 vaginal samples by Gram stain, by DNA-based techniques – like cloning and sequencing of amplified 16S rRNA-genes [13-15] and species specific PCR [12,15-18] – which make it possible to detect fastidious bacteria like *A. vaginae *[13,16,17] and by culture in combination with tDNA-PCR [20,21], which allows the rapid identification of large numbers of cultured isolates, including isolates from different *Lactobacillus *species [22]. Based on these findings, we propose refined criteria to categorize the status of the microflora of vaginal smears.

## Results

We studied the composition of the vaginal microflora of 515 vaginal swabs from a prospective cohort of 197 unselected pregnant women at three time points during pregnancy using i) Gram stain based grading according to modified Ison & Hay criteria [6] – which will be further denoted here as the criteria of Claeys, ii) culture in combination with molecular identification of cultured organisms by tDNA-PCR and iii) species specific PCR for *G. vaginalis *and *A. vaginae*.

**Figure 1:**
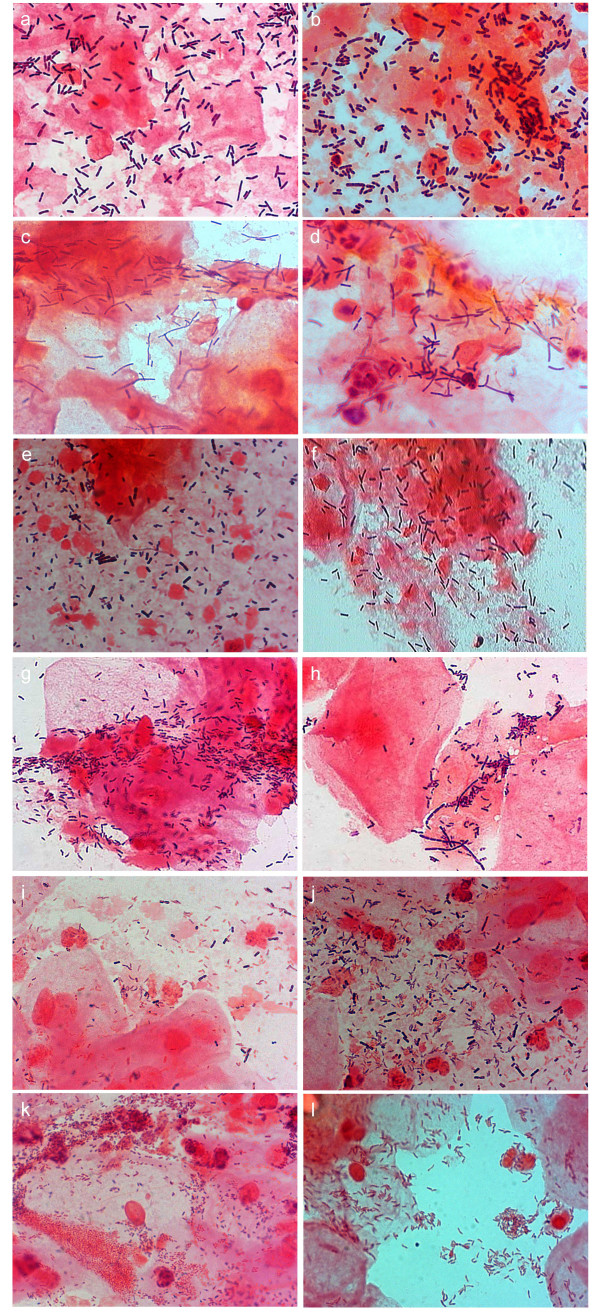
**Microscopic image (100 ×) of Gram-stained vaginal smears illustrating the different categories of vaginal microflora described:**. a, b: grade Ia, i.e. mainly *Lactobacillus crispatus *cell types, plump quite homogeneous lactobacilli. c, d: grade Ib, i.e. non-*L. crispatus *cell types, long or short, thin lactobacilli. e, f: grade Iab, i.e. containing mixtures of *L. crispatus *and non-*L. crispatus *cell types. g, h: grade I-like, i.e. irregular-shaped Gram positive rods. i, j: grade II, i.e. mixture of *Lactobacillus *cell types and bacterial vaginosis-associated bacteria (*Gardnerella*, *Bacteroides-Prevotella *and *Mobiluncus *cell types). k, l: grade III, i.e. bacterial vaginosis.

Detailed observation of the Gram stained vaginal smears in combination with specific PCR and tDNA-PCR based identification of cultured isolates led to subdivision of grade I samples and the recognition of a separate category, designated grade I-like: Grade I specimens were characterized as grade Ia when only *Lactobacillus crispatus *cell types (plump, mostly short rods) were present (Figure 1a – 1b), as grade Ib when only other *Lactobacillus *cell types were present (smaller or more elongated and less stained than in Ia smears)(Figure 1c – 1d) and as grade Iab when both *L. crispatus *and other lactobacilli were present (Figure 1e – 1f). Furthermore a number of samples were designated as grade I-like because of the presence of Gram positive rods, either quite small and short or otherwise irregularly shaped with clubbing, curved edges and irregular staining and often arranged like Chinese letters ('diphtheroid cell types') (Figure 1g – 1h). To corroborate that grade I-like samples indeed represent a separate class, cloning was carried out for two samples that had been categorized as grade I-like. For completeness, figures 1i – 1j represent grade II vaginal smears and figures 1k – 1l represent grade III vaginal smears.

### Comparison between Gram stain and culture

Using the criteria of Claeys, 162 vaginal smears were scored as grade Ia, 181 as grade Ib, 60 as grade Iab, 36 as grade I-like, 46 as grade II, 22 as grade III and eight as grade IV (Table 1).

We cultured 1108 isolates anaerobically out of the 515 vaginal swabs and identified these with tDNA-PCR. A total of 136 isolates remained unidentified, since no corresponding tDNA-PCR fingerprint could be found in the database or because no amplification was obtained. A total of 72 species were identified, of which 17 belonged to the genus *Lactobacillus *and six to the genus *Bifidobacterium *(Table 1). The most common species recovered from grade Ia, Ib and Iab specimens were lactobacilli. *L. crispatus *(87.0%) and *L. jensenii *(22.2%) were the most abundant bacteria in grade Ia samples, whereas *L. gasseri *(32.0%) and *L. iners *(39.8%) were the most frequently present species in grade Ib specimens. Grade I-like specimens were found to contain mostly bifidobacteria (54.9%) and *L. gasseri *(52.8), while *L. crispatus *was almost absent (2.8%). In 19.8% of grade I-like specimens bifidobacteria were present while lactobacilli were absent. Bifidobacteria were more frequent in grade I-like samples than in other samples (χ^2 ^= 120.6, p < 0.001, Table 2).

*L. crispatus *was present in 87.0% of grade Ia, 76.7% of grade Iab and 37.5% of grade IV samples but in less than 13.3% in all other grades. *L. crispatus *was the only *Lactobacillus *species that was linked to a single grade, namely grade Ia (χ^2 ^= 186.3, p < 0.001), while the other lactobacilli were more evenly distributed over all samples (Table 3, 4, 5, 6). *L. jensenii *was the second most abundant species in grade Ia (22.2%), but was also frequent in most other grades, for example in 47.8% of grade II. *L. vaginalis*, the third most abundant species in grade Ia (9.3%) was absent from grade III and present in less than 20% of all other grades. *L. gasseri *and *L. iners *were more abundant in grade Ib (32.0 and 39.8%), grade I-like (52.8 and 19.4%), grade II (54.3 and 26.1%) and grade III (9.1 and 31.8%) than in grade Ia (6.8 and 3.7%).

The most characteristic cultured organisms in grade II and grade III specimens were *G. vaginalis *(respectively 21.7% and 72.7%) (χ^2 ^= 120.6, p < 0.001, Table 7), *Actinomyces neuii *(respectively 6.5% and 9.1%), *Aerococcus christensenii *(respectively 4.3% and 22.7%), *A. vaginae *(respectively 4.3% and 13.6%), *Finegoldia magna *(respectively 2.2% and 9.1%) and *Varibaculum cambriense *(respectively 2.2% and 13.6%). These were virtually absent from grade I and grade IV, although *G. vaginalis *was present in approximately 2.0% of grade I samples. *L. jensenii *(47.8%) and *L. gasseri *(54.3%) were the most common lactobacilli in grade II specimens. Furthermore, whereas *L. crispatus *and *L. vaginalis *were never cultured from grade III specimens, *L. iners *(31.8%) was the lactobacillus mostly present in grade III. *Mobiluncus curtisii *and *Peptostreptococcus *sp. were cultured from grade III specimens only (both 4.5%). *Dialister *sp. (22.7%) and *Prevotella *spp. (22.6%) were frequently cultured from grade III specimens and only sporadically from other specimens.

The average number of species cultured per sample ranged from 1.5 for grade Ia specimens to 3.6 for grade III specimens (Table 8). Overall, the species diversity of the grade I-like category was higher (0.83) than that of the grade I subcategories (0.17, 0.21 and 0.30 for grades Ia, Ib, and Iab respectively) and comparable to that of the grade II category (0.76). The grade III category had the highest species diversity (1.50) (Table 8).

### Comparison between Gram stain and species specific PCR for *Gardnerella vaginalis *and *Atopobium vaginae*

The series of 515 vaginal samples were analyzed by PCR with 16S rRNA gene based primers specific for *A. vaginae *and 16S–3S spacer primers specific for *G. vaginalis*.

After amplification with the ato167f *A. vaginae *primer set, respectively 14.7% of grade I, 8.3% of grade I-like, 28.3% of grade II, 86.4% of grade III and 12.5% of grade IV samples showed an amplicon. The percentage of positive samples for *G. vaginalis *specific PCR was respectively 28.9%, 19.4%, 47.8%, 86.4% and 12.5%.

The simultaneous presence of *A. vaginae *and *G. vaginalis *in a vaginal swab specimen had an accuracy of 90% [95% CI: 86–92%], a sensitivity of 82% [95% CI: 59–94%], a specificity of 90% [95% CI: 87–92%], a positive predictive value of 26% [95% CI: 17–39%] and a negative predictive value of 99% [95% CI: 98–100%] in assessing bacterial vaginosis (defined as a grade III smear).

### Comparison between culture and cloning of grade I-like samples

Cloning of two grade I-like samples from trimesters 1 and 2 of the same patient, revealed the presence of *Bifidobacterium breve *(respectively 33.1 and 53.5%), *Lactobacillus delbrueckii *(64.8 and 13.3%) and *L. gasseri *(2.1 and 33.1%) clones. This was in agreement with the culture results which revealed the presence of *B. breve *in both trimesters, *L. delbrueckii *only in the first and *L. gasseri *only in the second.

In general, grade I-like samples were found by culture to contain more frequently *Bifidobacterium *(19/36 samples) and more different *Bifidobacterium *species (6) than samples from all other categories. Of the *Bifidobacterium *species, *B. breve *was most clearly associated with grade I-like, grade II and grade III.

## Discussion

### The importance of correct diagnosis of bacterial vaginosis and of more detailed characterization of the vaginal microflora

Although not causing a vaginal inflammatory response, bacterial vaginosis is considered to be the most common cause of vaginitis in pregnant and non-pregnant women and prevalences between 4.9% and 36.0% have been reported from European and American studies [23]. Several studies suggest the possibility that bacterial vaginosis increases the risk of acquiring HIV [24,25] and that the bacterial flora associated with bacterial vaginosis increases genital-tract HIV shedding [26]. A recent meta-analysis by Leitich *et al*. [27] established an odds ratio of 8 for preterm birth in association with bacterial vaginosis during early pregnancy. Spontaneous preterm birth occurs in 7–11% of pregnancies but accounts for three quarters of perinatal morbidity and mortality and for half of long term neurological impairment in children [28,29].

Bacterial vaginosis is characterized by the replacement of the normal vaginal microflora of lactobacilli by *Gardnerella vaginalis *and anaerobic organisms. Recently, different groups showed that the strict anaerobe *Atopobium vaginae *is another organism that is strongly associated with bacterial vaginosis [12,13,16,17]. The association between *A. vaginae *and bacterial vaginosis might help explain why some women suffer from recurrent bacterial vaginosis. For example, a recent study pointed to great *in vitro *efficacy of metronidazole, since this antibiotic inhibited growth of 99% of the vaginal isolates from bacterial vaginosis samples [30], but most likely overlooked the fastidious metronidazole resistant *A. vaginae*, shown in this study to be present in 86.4% of bacterial vaginosis samples when detected with species specific PCR.

Given the possibility that certain not yet characterized subgroups within the presumably heterogenic clinical entity of women with bacterial vaginosis could identify a group at higher risk for preterm birth than women with bacterial vaginosis as a whole and that adequate treatment of women from this higher risk group may allow for more targeted preterm birth prevention, better understanding of the composition and dynamics of the vaginal microflora and accurate diagnosis of bacterial vaginosis are warranted. Also, our data indicate that refined characterization of vaginal microflora may be necessary for more accurate interpretation of the results of clinical studies. For example, thus far *Atopobium vaginae *has been overlooked in clinical studies and furthermore, the fact that different *Lactobacillus *species may confer different strengths of colonisation resistance [10,11] has not been taken into account, partly because most laboratories lack the access to rapid and accurate methods for the identification of lactobacilli to the species level. In other words, several studies concerning the relation between the status of the vaginal microflora and different gynecologic and obstetric diseases and their treatments thus far may have reached biased conclusions due to insufficiently precise characterization of the microflora.

### Criteria for microbiological categorization of vaginal microflora status

Spiegel *et al*. [31] defined a scoring system based on some of the bacterial cell types that can be seen in Gram stained smears of vaginal secretion. This was later refined by Nugent et al. [1], who provided a scoring system that evaluates the changes in vaginal microflora, from the normal condition to bacterial vaginosis status, as a continuum. Although the Nugent criteria have gained wide acceptance for the evaluation of the condition of the vaginal microflora [2,32], further refinement is warranted for several reasons. First, no definite criteria have been described to distinguish the *Lactobacillus *cell types from the *Gardnerella *and *Bacteroides*-*Prevotella *cell types. In practice and in our experience, 'morphotypes' are often difficult to assign to one of these groups. Also, some genera and species that are clearly associated with bacterial vaginosis, like *Peptostreptococcus *spp. [32] and *A. vaginae *[12,17,13] are not included in the Nugent score. Furthermore, Forsum *et al*. [2] found major discrepancies in scoring when the lactobacillary cell types were few in number and Larsson *et al*. [33] reported several problems in the interpretation of smears. For example, using the Nugent criteria, the presence of different *Lactobacillus *cell types in smears from patients with bacterial vaginosis can lead to assignation to grade II, whereas patients without bacterial vaginosis but with smears with more than 300–500 pleomorphic *Lactobacillus *cells may be regarded as containing *G. vaginalis*, also because some of these cells are very small. Additionally, the Nugent scoring system conflates women with potentially very different vaginal microflora in a single category [3].

In this study, the clinical microbiologist (GC) could not grade some of the smears due to the presence of cell types not scored in the system developed by Nugent [1] and classified these samples as grade I-like. Further detailed observation lead to the splitting up of grade I samples into subcategories designated grade Ia, grade Ib and grade Iab. After blind grading of the vaginal smears into grades Ia, Ib, Iab, I-like, II, III and IV, this classification was compared with the culture results and with species specific PCRs.

### Grade Ia and Iab: Agreement with the presence of *L. crispatus*

From this comparison it became obvious that it is possible to recognize the presence of *L. crispatus *by means of Gram stain, since this species was cultured in 81.9% of the grade Ia samples and 76.7% of the grade Iab samples. Nevertheless, *L. crispatus *was not cultured from 21 of the 162 grade Ia samples. This may be explained by the fact that *L. crispatus *is not as easily cultured as other lactobacilli. Indeed, *L. crispatus *colonies were quite often observed as satellites of other bacteria and in some cases no growth at all was observed in samples with numerous *L. crispatus*-like lactobacilli on Gram stain. Using non culture dependent t-RFLP-analysis (data not presented) the Ia samples negative for *L. crispatus *culture were tested and 16 were positive for *L. crispatus*, bringing the agreement between Gram stain grading as grade Ia and the presence of *L. crispatus *to 96.9%. Similarly, when taking into account t-RFLP-analysis results, the agreement between categorization as grade Iab and t-RFLP-analysis positive for *L. crispatus *was 92.9% whereas *L. crispatus *was detected by t-RFLP-analysis only in 27.3%, 20.0%, 22.5% and 0% for grades Ib, I-like, II and III, respectively. These results indicate that – for a trained microbiologist – it is possible to recognize *L. crispatus *bacteria upon cell morphology, a finding that is of importance since this species is clearly associated with healthy microflora, and possibly better ensures stable healthy microflora than other lactobacilli [9]. Samples were scored as grade Ib when no *L. crispatus *cell types were observed, but other *Lactobacillus *cell types were predominant. The agreement with culture results was high: only 13.3% contained *L. crispatus *upon culture, whereas *L. gasseri*, *L. iners*, and *L. jensenii *were present in respectively 32.0, 39.8, and 24.3% of the grade Ib samples. These were clearly grade I samples since bacterial vaginosis-associated organisms were mostly absent.

### The colonisation resistance conferred differs between *Lactobacillus *species

Overall the frequency of isolation of all *Lactobacillus *species together was comparable for the different grades in our population, since lactobacilli were cultured from 96.9% of grade Ia, 94.5% of grade Ib, 96.7% of grade Iab, 78.9% of grade I-like, 93.5% of grade II, 59.1% of grade III and 62.5% of grade IV samples. This is in agreement with previous reports [32,34]. However, we observed a clear difference with regard to the *Lactobacillus *species frequency distribution for the different grades. While *L. crispatus*, known as a strongly H_2_O_2_-producing species [7,8], was cultured from 87.0% of grade Ia specimens, it was absent in grade III specimens and only present in 2.8% of grade I-like specimens. In contrast, *L. iners*, reported as a weakly H_2_O_2_-producing species [7,8], was present in only 3.7% of Ia specimens but in 39.8% of grade Ib and 31.8% of grade III specimens. Whether it is the hydrogen peroxide production by *L. crispatus *that confers colonisation resistance remains a matter of debate, since a correlation between the presence of hydrogen peroxide production and the type of vaginal microflora was found by some [35], though not by others [36]. Possibly other species specific characteristics, present in *L. crispatus*, but absent in species like *L. gasseri *and *L. iners*, confer colonisation resistance. It has also been hypothesized that the onset of perturbation leading to bacterial vaginosis may be due to competition between *Lactobacillus *species [36], a situation possibly reflected by grade Iab specimens.

### Grade I-likes: a separate category of vaginal microflora status

A number of samples were initially difficult to score because the predominant cell types could not be categorized as *Lactobacillus*, *Gardnerella*, *Bacteroides*-*Prevotella *or *Mobiluncus *cell types. These samples were considered as belonging to a separate category because of the presence of Gram positive rods, either quite small and short or otherwise irregularly shaped with clubbing, curved edges and irregular staining and often arranged like Chinese letters ('diphtheroid cell types'). Since it is likely that most microbiologists would score this cell type as '*Lactobacillus*-like' and that therefore it would be scored in most cases as grade I, we designated it as 'grade I-like'. Culture and species specific PCR confirmed that indeed these samples represent a separate kind of vaginal microflora. This is reflected by the increased species diversity of 0.83, which is much higher than that for grades Ia, Ib and Iab (0.15–0.30) and which is comparable to that of grade II (0.76), but even more so by the virtual absence of *L. crispatus *(cultured from only one of 36 samples) as well as of *G. vaginalis *and *A. vaginae *(cultured from respectively 1 and 0 samples) and the presence of *Bifidobacterium *spp. in 19 of 36 samples, a much higher prevalence than in samples from all other grades. This was confirmed by cloning of two grade I-like samples, which contained only *L. delbrueckii*, *L. gasseri *and *B. breve*.

Rosenstein *et al*. [34] mentioned a category of vaginal smears with aberrant morphology, which they designated as grade I revertants. At first sight, their category shows resemblance with the category we describe here as I-like, because of low numbers of *G. vaginalis *and increased numbers of bifidobacteria, but on the other hand they reported even more bifidobacteria in their grade II and grade III samples and they designated this category as grade I revertants because the vaginal microflora of all 41 women with such smears reverted to grade I, which was not the case in our study (data to be presented elsewhere).

Importantly, since Gram stain based categorizing can result in the interpretation of grade I-like samples as genuine grade I samples (whereof their designation), this class of samples may jeopardize – and probably has done so in the past – the interpretation of the results of clinical studies.

### Grade II: a microbiologically intermediate stage between healthy microflora and bacterial vaginosis

Our results confirm that grade II samples represent a microbiologically clearly intermediate status between grade I and III. *L. crispatus *is still present in 10.9% of the samples (compared to 59.0% of grade I and 0% of grade III samples), whereas the number of samples with *L. gasseri *(54.3%) is increased compared to grade I (21.3%) and grade III (9.1%). Species diversity of grade II is intermediate between that for grade I and grade III and species typically associated with bacterial vaginosis, like *A. neuii*, *A. christensenii*, *A. vaginae*, *B. ureolyticus*, *F. magna*, *G. vaginalis*, *Peptoniphilus sp*. and *V. cambriense*, are present, but again in a lower number of samples than in grade III specimens.

### Grade III: Characterization of bacterial vaginosis -related organisms

The following species are generally considered as bacterial vaginosis related anaerobe organisms: *Anaerococcus (Peptostreptococcus) tetradius*, *A. (Peptostreptococcus) vaginalis*, *Atopobium vaginae*, *Bacteroides ureolyticus*, *Finegoldia (Peptostreptococcus) magna*, *G. vaginalis*, *Gemella (Peptostreptococcus) morbillorum*, *Mobiluncus curtisii*, *Mycoplasma hominis*, *Peptoniphilus *sp., *Peptostreptococcus *sp., *Prevotella bivia*, *Prevotella ruminicola *and *Prevotella *sp. [37,38]. Using tDNA-PCR we were able to identify 87.8% of the cultured isolates to the species level and found our results to be largely in agreement, but in addition we cultured *Actinomyces neuii*, *Aerococcus christensenii*, *Dialister *sp. and *Varibaculum cambriense*, whereas *Mobiluncus *spp., *Mycoplasma hominis *and *Ureaplasma urealyticum *were not or only sporadically cultured from grade II and grade III specimens. The absence of the latter species in our study can be explained by the fact that we did not use the specific culture methods for these fastidious organisms.

In this study we confirmed the strong association, as established previously [12,13,17], between *A. vaginae *and bacterial vaginosis.

## Conclusion

In summary, our characterization of the vaginal microflora by means of detailed Gram stain interpretation and by culture in combination with genotypic identification helps to refine our understanding of normal and disturbed vaginal microflora. We showed that *L. crispatus *can be recognized as such on Gram stain, we established the existence of a separate additional category, characterized by the absence of *L. crispatus *and the abundance of bifidobacteria and we confirmed the association of *Atopobium vaginae *with bacterial vaginosis.

These data have implications for the basic understanding of the vaginal microflora and bacterial vaginosis; in addition, they may add to the value of Gram smear based diagnosis of bacterial vaginosis because of better defined Gram stain criteria.

## Methods

### Study population and sample collection

A total of 515 vaginal swabs were collected by sampling 197 pregnant women attending our out-patient clinic, each at three time points during pregnancy (respectively 197, 171 and 147 first, second and third trimester samples were collected). The swabs were obtained during the first, second and third pregnancy trimester, at mean gestational ages of 9.1 +/- 3.2 weeks, 20.4 +/- 2.3 weeks and 32.2 +/- 1.7 weeks, respectively.

Sampling was carried out by insertion of three sterile cotton swabs into the vaginal vault, after placement of a non-lubricated speculum. The swabs were rotated against the vaginal wall at the midportion of the vault and were carefully removed to prevent contamination with microflora of the vulva and introitus. The first swab was used to prepare a smear on a glass slide for the purpose of grading according to the criteria of Claeys (this study). The second swab was returned to a sterile tube (Copan, Brescia, Italy), for the purpose of DNA-extraction (dry swab). The third swab was placed into Amies transport medium (Nuova Aptaca, Canelli, Italy) for anaerobic culture. The unstained smear and both swabs were sent to the microbiology laboratory and were processed within 4 hours.

### Grading of slides

Smears were dried, Gram stained (Mirastainer, Merck-Belgolabo, Overijse, Belgium) and examined under oil immersion at a magnification of 1000. Gram stained smears from vaginal swabs were all scored by one clinical microbiologist (GC) according to Ison & Hay criteria [5,6]: samples were categorized as grade I when only *Lactobacillus *cell types (large Gram positive rods) were present, as grade II (intermediate) when both *Lactobacillus *and *Gardnerella *or *Bacteroides-Prevotella *cell types were present, as grade III (bacterial vaginosis) when *Lactobacillus *cell types were absent and only *Gardnerella*,*Bacteroides-Prevotella *or *Mobiluncus *cell types were present and as grade IV when Gram positive cocci were predominantly present. Further subdivision of grade I samples into categories Ia, Iab and Ib and the description of a separate category, designated grade I-like, is presented in the Results section.

### Culture and identification of cultured isolates by tDNA-PCR

For 515 specimens collected from 197 women, the swab on Amies transport medium was streaked onto Schaedler agar enriched with 5% sheep blood, vitamin K, hemin and sodium pyruvate (Becton Dickinson, Franklin Lakes, NJ) and incubated anaerobically at 37°C upon arrival at the microbiology laboratory. After 4 days of incubation, all the isolates with different colony morphology were selected for identification. DNA was extracted by simple alkaline lysis: one colony was suspended in 20 μl of 0.25% sodium dodecyl sulfate-0.05 N NaOH, heated at 95°C for 15 min and diluted with 180 μl of distilled water. tDNA-PCR and capillary electrophoresis were carried out as described previously [20,22]. The species to which each isolate belonged was determined by comparing the tDNA-PCR fingerprint obtained from each isolate with a library of tDNA-PCR fingerprints obtained from reference strains, using an in-house software program [20]. The library of tDNA-PCR fingerprints is available at our website [39] and the software can be obtained upon request.

### DNA extraction of vaginal swab samples

For DNA extraction from the dry vaginal swabs, the QIAamp DNA mini kit (Qiagen, Hilden, Germany) was used according to the manufacturer's recommendations, with minor modifications, as described previously [13]. DNA-extracts were stored at -20°C and were used for the purpose of species specific PCR and cloning experiments.

### Species specific PCR for *Gardnerella vaginalis*

*G. vaginalis *species specific primers as designed by Zariffard *et al*. (G_Z_) [19] were used. Briefly, a 20 μl PCR mixture contained respectively 0.05 and 0.4 μM primers, 10 μl of Promega master mix (Promega, Madison, WI), 2 μl of Qiagen DNA-extract of the samples and distilled water. Thermal cycling with G_Z _primers consisted of an initial denaturation of 10 min at 94°C, followed by 50 cycles of 5 s at 94°C, 45 s at 55°C and 45 s at 72°C, and a final extension of 10 min at 72°C. Five microliter of the amplified product was visualized on a 2% agarose gel.

### Species specific PCR for *Atopobium vaginae*

A primer set that allowed amplification of the 16S rRNA gene of *A. vaginae *and that lacked homology with non-target bacteria was used as described earlier [13]. Briefly, a 10 μl PCR mixture contained 0.2 μM each of the primers ato167f (5' GCGAATATGGGAAAGCTCCG) and ato587r (5' GAGCGGATAGGGGTTGAGC), 5 μl of Promega master mix (Promega, Madison, WI), 1 μl of Qiagen DNA-extract of the samples and distilled water. Thermal cycling consisted of an initial denaturation of 5 min at 94°C, followed by three cycles of 1 min at 94°C, 2 min at 58°C and 1 min at 72°C, followed by 35 cycles of 20 sec at 94°C, 1 min at 58°C and 1 min 72°C, with a final extension of 10 min at 72°C, and cooling to 10°C. Five microliter of the amplified product was visualized on a 2% agarose gel. The primers amplified a DNA-fragment of 420 base pairs from *A. vaginae *and showed no cross reactivity to other organisms, including *A. rimae *and *A. parvulum *(data not presented).

### Cloning of amplified mixtures of 16S rDNA

Cloning and sequencing was carried out largely as described previously [13]. However, to increase the amplification efficiency of the 16S rRNA-genes of *G. vaginalis *and bifidobacteria the following mixture of primers (0.1 μM each) was used for the initial amplification of the samples prior to cloning: primers 10 f (5' AGTTTGATCCTGGCTCAG), 534r (5' ATTACCGCGGCTGCTGG) and GV10f (5' GGTTCGATTCTGGCTCAG).

### Statistical analysis

The Simpson's Diversity Index was calculated as D = 1-∑ (n/N)^2 ^where n is the number of isolates of a particular species and N is the total number of isolates. Chi square analyses were carried out using the statistical software package SPSS v.11.0.

## Authors' contributions

RV, GC, GV, EDB and MV participated in the development of the study design, the analysis of the study samples, the collection, analysis and interpretation of the data, and in the writing of the report. HV and MT participated in the development of the study design, the collection of the study samples, the collection, analysis and interpretation of the data, and in the writing of the report. LVS and CDG participated in the analysis of the study samples. All authors read and approved the final manuscript.

## Figures and Tables

**Table 1:** Detailed composition of the vaginal microflora of 515 vaginal swab samples, as determined by culture and tDNA-PCR based identification

**Species**	**Vaginal smears graded by Gram stain**							
**Grade**	**Ia**	**Ib**	**Iab**	**I-like**	**II**	**III**	**IV**	**Total**
**Number of samples **	**162**	**181**	**60**	**36**	**46**	**22**	**8**	**515**
***Lactobacillus *spp.**								
*Lactobacillus crispatus*	87.0^a^	13.3	76.7	2.8	10.9		37.5	42.7
*Lactobacillus jensenii*	22.2	24.3	43.3	13.9	47.8	18.2	12.5	26.8
*Lactobacillus gasseri*	6.8	32.0	25.0	52.8	54.3	9.1	25.0	25.6
*Lactobacillus iners*	3.7	39.8	8.3	19.4	26.1	31.8		21.2
*Lactobacillus vaginalis*	9.3	12.7	15.0	11.1	6.5		20.0	1.7
								
*Lactobacillus casei*		1.1	1.7	2.8	2.2	4.5	12.5	1.4
*Lactobacillus coleohominis*	1.2	5.5	5.0		2.2			3.1
*Lactobacillus delbrueckii*		0.6		5.6	2.2			0.8
*Lactobacillus fermentum*	0.6	1.1	3.3			4.5		1.2
*Lactobacillus kalixensis*		0.6						0.2
*Lactobacillus mucosae*					4.3			0.4
*Lactobacillus nagelii*					2.2			0.2
*Lactobacillus oris*					4.3			0.4
*Lactobacillus pontis 94%*		0.6						0.2
*Lactobacillus reuteri*		1.7	1.7	5.6				1.2
*Lactobacillus rhamnosus*		0.6		8.3	4.3	4.5	12.5	1.6
*Lactobacillus salivarius*		0.5						0.2
***Bifidobacterium *spp.**								
*Bifidobacterium biavatii*		0.6		5.6			12.5	0.8
*Bifidobacterium bifidum*		0.6		2.8		4.5		0.6
*Bifidobacterium breve*		0.6		25.0	10.9	9.1		3.3
*Bifidobacterium dentium*	0.6			8.3		4.5		1.0
*Bifidobacterium longum*	0.6	0.6		5.6		4.5		1.0
*Bifidobacterium *sp.		0.6		5.6				0.6
**Bacterial vaginosis-related anaerobe organisms**								
*Actinomyces neuii*					6.5	9.1		1.0
*Aerococcus christensenii*					4.3	22.7		1.4
*Anaerococcus tetradius*^b^					2.2			0.2
*Anaerococcus vaginalis*^b^				2.8				0.2
*Atopobium vaginae*		0.6			4.3	13.6		1.2
*Bacteroides ureolyticus*	0.6			2.8	2.2	9.1		1.0
*Dialister *sp.						22.7	12.5	1.2
*Finegoldia magna*^b^	0.6	0.6			2.2	9.1		1.0
*Gardnerella vaginalis*	1.2	2.8	1.7	2.8	21.7	72.7		6.8
*Gemella morbillorium*^b^					2.2			0.2
*Mobiluncus curtisii*						4.5		0.2
*Mycoplasma hominis*					4.3			0.4
*Peptoniphilus *sp.^b^	3.1	1.7		2.8	6.5	9.1		2.7
*Peptostreptococcus *sp.						4.5		0.2
*Prevotella bivia*		0.6		2.8		13.6		1.0
*Prevotella ruminicola*						4.5		0.2
*Prevotella *sp.						4.5		0.2
*Varibaculum cambriense*					2.2	13.6		0.8
**Other species**								
*Actinomyces europaeus *96%	0.6							0.2
*Actinomyces urogenitalis*						4.5		0.2
*Arcanobacterium bernardiae*	0.6							0.2
*Arthrobacter albus*					4.3			0.4
*Atopobium parvulum*						4.5		0.2
*Clostridium colicanis*			3.3					0.4
*Clostridium *sp.						4.5		0.2
*Corynebacterium amycolatum*				2.8				0.2
*Corynebacterium coyleae*		0.6						0.2
*Corynebacterium pseudogenitalium*	0.6							0.2
*Corynebacterium *sp.		0.6			4.3			0.6
*Enterococcus faecalis*	2.5	4.4	3.3		2.2	4.5		3.1
*Escherichia coli*		0.6	3.3	2.8		9.1		1.2
*Haemophilus influenzae*					2.2			0.2
*Helcococcus *sp.						4.5		0.2
*Pediococcus pentosaceus*				2.8		4.5		0.4
*Propionibacterium acnes*	1.9							0.6
*Propionibacterium avidium*				2.8				0.2
*Serratia *sp.		0.6						0.2
*Staphylococcus aureus*	0.6	0.6			2.2			0.6
*Staphylococcus capitis*		0.6						0.2
*Staphylococcus epidermidis*	1.2	5.5	1.7	5.6	4.3			3.3
*Staphylococcus haemolyticus*			1.7		2.2			0.4
*Staphylococcus hominis*		1.1		2.8	2.2			0.8
*Streptococcus agalactiae*	1.9	4.4	1.7	11.1		4.5	75.0	4.5
*Streptococcus anginosus *group	3.1	3.9	1.7	5.6	4.3	9.1		3.7
*Streptococcus gallolyticus*			1.7					0.2
*Streptococcus mitis*				2.8	2.2			0.4
*Streptococcus salivarius*	0.6	1.7						0.8
*Veilllonella atypica*	1.2	0.6		2.8				0.8
*Veillonella *sp.	0.6	1.1			2.2			0.8

^a ^Numbers represent percentage of samples from which the species was cultured.

^b ^Formerly known as *Peptostreptococcus*.

**Table 2:** Presence of *Bifidobacterium *spp. in grade I like samples versus other samples.

***Bifidobacterium *spp.**	**Grade I-like**	**Other grades**	**Total**
Cultured	19	18	37
Not cultured	17	461	478
Total	36	479	515

**Table 3:** The presence of *Lactobacillus *species in grade Ia and grade Ib samples.

	**Grade Ia**	**Grade Ib**	**Total**
**Total**	**162**	**181**	**343**
*L. crispatus*	141	24	165
*L. jensenii*	36	44	80
*L. gasseri*	11	58	69
*L. iners*	6	72	78

**Table 4:** Presence of *L. gasseri *or *L. iners *in grade II and grade III samples versus presence in other samples.

***L. gasseri *or *L. iners***	**Grades II and III**	**Other grades**	**Total**
Cultured	41	184	225
Not cultured	27	263	290
Total	68	447	515

**Table 5:** Number of samples with lactobacilli in grade Ia versus the other grades.

**Species**	**Grade Ia**	**Other grades**	**Total**
***L. crispatus***	185	35	220
***L. jensenii***	36	102	138
***L. gasseri***	11	123	132
***L. iners***	6	103	109

**Table 6:** Number of samples with lactobacilli in grade I versus the other grades.

**Species**	**Grades I a + Iab + Ib**	**Other grades**	**Total**
***L. crispatus***	211	9	220
***L. jensenii***	106	32	138
***L. gasseri***	84	48	132
***L. iners***	83	26	109

**Table 7:** Presence of *G. vaginalis *in grade II and grade III samples versus presence in other samples.

***G. vaginalis***	**Grades II and III**	**Other grades**	**Total**
Cultured	26	9	35
Not cultured	42	438	480
Total	68	447	515

**Table 8:** Numbers of species cultured per patient and species diversity indices

**Grade**	**Number of species cultured**		**Diversity**	
	**Average**	**Range**	**Number of Species/Number of Samples (Index)**	**Simpson's Diversity Index**

Ia	1.5	1–6	27/162 (0.17)	0.6
Ib	1.7	1–7	38/181 (0.21)	0.9
Iab	2.0	1–7	18/60 (0.30)	0.8
I-like	2.3	1–8	30/36 (0.83)	0.9
II	2.7	1–6	35/46 (0.76)	0.9
III	3.6	2–8	33/22 (1.50)	0.9
IV	2.0	1–3	5/8 (0.63)	0.8
